# Geophonino-W: A Wireless Multichannel Seismic Noise Recorder System for Array Measurements

**DOI:** 10.3390/s19194087

**Published:** 2019-09-21

**Authors:** Juan Luis Soler-Llorens, Juan José Galiana-Merino, José Juan Giner-Caturla, Sergio Rosa-Cintas, Boualem Youcef Nassim-Benabdeloued

**Affiliations:** 1 Department of Earth Sciences and Environment, University of Alicante, Crta. San Vicente del Raspeig, s/n, 03080 Alicante, Spain; jj.giner@ua.es; 2 Department of Physics, Systems Engineering and Signal Theory, University of Alicante, Crta. San Vicente del Raspeig, s/n, 03080 Alicante, Spain; juanjo@dfists.ua.es (J.J.G.-M.); boualem.youcef.nassim.benabdel@upc.edu (B.Y.N.-B.); 3 University Institute of Physics Applied to Sciences and Technologies, University of Alicante, Crta. San Vicente del Raspeig, s/n, 03080 Alicante, Spain; sergio.rosacintas@ua.es; 4 Department of General Didactics and Specific Didactics (Faculty of Education), University of Alicante, Crta. San Vicente del Raspeig, s/n, 03080 Alicante, Spain; 5 Department of Fluid Mechanics, Polytechnic University of Catalonia, C. Jordi Girona, 31, 08034 Barcelona, Spain

**Keywords:** seismic data acquisition, seismic noise measurement, Arduino Due, ambient-noise recorder, customized hardware, seismic signal conditioning circuit

## Abstract

The characterization of soil is essential for the evaluation of seismic hazard, because soil properties strongly influence the damage caused by earthquakes. Methods based on seismic noise are the most commonly used in soil characterization. Concretely, methods based on seismic noise array measurements allow for the estimation of Rayleigh wave dispersion curves and, subsequently, shear-wave velocity profiles. The equipment required for the application of this technique is usually very expensive, which could be a significant economic challenge for small research groups. In this work, we have developed a wireless multichannel seismic noise recorder system (Geophonino-W), which is suitable for array measurements. Each station includes a microcontroller board (Arduino), a conditioning circuit, an Xbee module, an SD card, and a GPS module. Several laboratory tests were carried out in order to study the performance of the Geophonino-W: A frequency response test (impulse response and noise); synchronization test; and battery duration test. Comparisons of Geophonino-W with the commercial systems and field measurements were also carried out. The estimated dispersion curves obtained using the proposed system were compared with the ones obtained using other commercial equipment, demonstrating the effectiveness of Geophonino-W for seismic noise array measurements. Geophonino-W is an economic open-source and hardware system that is available to any small research group or university.

## 1. Introduction

The damage produced in different places by the occurrence of an earthquake is not only related to the energy released. The level of damage at each site is closely linked to what is called the “site effect”. This effect can significantly increase the seismic shaking at the surface of the site, especially when it is constituted by soft sediments [1,2,3]. The seismic wave amplification is determined by the physical soil characteristics. Therefore, subsoil characterization is mandatory in the evaluation of seismic hazards in urban areas, because it provides information about the site effects when an earthquake occurs. The determination of the characteristics of subsoil could be conducted through geophysical (i.e., seismic, electric, and magnetic methods) and geotechnical methods (i.e., a borehole test, dynamic penetration test, and standard penetration test). In general, both methods can be very expensive. Furthermore, geotechnical methods are very invasive, and they are not suitable for application in urban areas. A brief revision of seismic methods will be addressed in Section 4, as they are the methods used in this work. 

Most seismic methods are based on array measurements, in which the analysis of signals recorded simultaneously by a set of sensors provides information about the propagation characteristics of surface waves (velocity-frequency response or dispersion curve) in analyzed soil. Respecting the array configuration in the field, the minimum requirements are three or more sensors that record simultaneously and at least one data acquisition recorder system [4]. Usually, the connection between the sensors and recorder system is established using wires, but wireless communication has begun to be employed in the last few years. A good solution for the communication between several sensors in the field is the implementation of a wireless sensor network (WSN). These seismic recorder systems are usually expensive and could pose a huge economic challenge, especially for a small research groups. A typical wired recorder system, suitable for seismic noise array measurements, acquired in 2017 by the University of Alicante, costs around 5000 € (taxes excluded), excluding sensors. This situation can occur especially in countries with limited economic resources, which is especially critical when the region presents a high seismic risk. This drawback might be minimized by the use of open-source and open-hardware equipment, which would reduce the cost (compared with other wireless systems).

The design and development of a highly customized WSN has become the standard in many researches. This has occurred for several reasons: (1) The continuous development of new devices that can be connected to several controllers and the addition of new functions to them; (2) the breakthrough of new low-consumption communication systems; and (3) the publication of open-source libraries, which allow for the simplification of the handling of these communications systems. A WSN is a system comprised of radio frequency (RF) transceivers, sensors, microcontrollers and power sources [5]. Highly customized WSNs have been applied in agriculture [5], food engineering [6], volatile chemical sensing [7], air pollution monitoring systems [8], logistics [9] and other research areas. WSNs can be applied in diverse environments, and they provide several advantages compared with wired connections systems, such as their small size, low cost, flexibility, low power consumption, distributed intelligence, scalability, environmentally friendly operation and ease of operation [6].

Over the last few years, several researchers have developed their own seismic recorder systems, some of which are based on highly customized WSNs. In 2010, Picozzi et al. [10] designed and implemented a wireless seismic recorder system (GFZ-WISE), which was originally developed for earthquake early warning systems. In this case, the developed system consumed a lot of energy, so it required a main power supply or solar panels. Furthermore, seismic noise array measurements require a strict synchronization among all the stations, which was not explicitly considered in this system [11].

Another related work was that of Fischer et al. [12], published in 2012, in which a centralized early warning system was presented. Afterwards, Lopes Pereira et al. [13] developed a WSN for monitoring volcano-seismic signals in 2014. Relatedly, Peci et al. [14] proposed an embedded Advanced Risk Machine (ARM) system for volcano monitoring in remote areas in 2014. Dai et al. [11] published, in 2015, a cableless geophone unit for passive surface wave surveys. In this case, the developed system was based on the ARM and the analog of the digital converter, ADS1251. This ARM supports a wireless communication unit, but it has not been detailed in this work. Moreover, the ADS1251 should be band-limited in order to prevent aliasing of the input signals [15].

The most recent works related to the development of wireless seismic recorder systems have been achieved between the years, 2017 and 2019. Jamali-Rad et al. [16] proposed a wireless network for seismic applications, based on the internet of things. An appropriate network architecture and data coordination was used to acquire seismic data of interest to the energy industry. However, this was not an open project, so it could not be reproduced. In the work of Martinez et al. [17], a WSN for investigating glacier stick-slip motion was presented. It was an innovative work for wireless extraction of borehole data. 

Finally, Boxberguer et al. [18] presented a new instrumental design for seismic data acquisition and processing (MPwise). While most of the components were off-the-shelf, the digitizer board was developed by GFZ Potsdam, and its design was not published, which made the reproduction of the system difficult. The most recent research work related to the development of seismic noise recorder systems was published in 2019 [19]. It implements a data quality real-time monitoring system, and it uses a dynamic frequency selection technology and a dynamic power management technology to design an energy-efficient system. It is, again, a work whose reproducibility is non-viable, because the detailed PCB design and the source code implementation are not published.

The aim of this paper is to present a new wireless multichannel seismic noise recorder system (Geophonino-W), based on a WSN and developed with open-source hardware (OSHW) and open-source software (OSS). In the developed system, the radio frequency transceivers used are Xbee modules, the sensors are geophones, the microcontroller is the Arduino Due board and the power source is a lithium ion battery, which powers the whole system. 

In order to validate the developed system, experimental measurements have been carried out at several sites, where the dispersion curve obtained with the f-k method was previously calculated using commercial equipment. The comparison of the estimated dispersion curves obtained using the commercial equipment and the ones obtained using the developed Geophonino-W system shows a good agreement. These results prove the right performance of the developed system in recording seismic noise data. 

In this paper, we first thoroughly describe the hardware parts of the developed system and then explain, in detail, all of the implemented software. In this way, we share a complete reproducible system, which is especially important in custom hardware or software publications [20]. Furthermore, all the necessary materials have been attached to this paper as a supplementary material and they have been shared through GitHub, one of the most popular sites for collaboration on OSS and OSHW and the largest open-source community in the world [21]. The shared material allows any researcher to assemble their own Geophonino-W system and use it for research or educational purposes.

This paper is structured as follows: A detailed description of the hardware and software of Geophonino-W is presented in Section 2, which is followed by several laboratory tests in Section 3, where the functionality of the Geophonino-W is analyzed. In Section 4, a brief overview of the literature is presented, followed by a field test to validate the Geophonino-W, where the dispersion curves obtained using Geophonino-W are compared with the ones obtained using commercial equipment. The paper concludes with an analysis of the results and conclusions in Section 5.

## 2. System Development and Implementation

### 2.1. General

The main objective of this work is to develop an open-source wireless acquisition system suitable for seismic noise array measurements. From a deployment point of view, some situations, in which the use of a wireless connection would be more suitable, are as follows:

- When accessibility to the desired location of the sensors is complicated, and the whole setup has to be established in-situ.
- In array measurements, when the deployment of several sensors, especially those with large apertures, may make the connection of the sensors with the recorder extremely difficult and even more difficult, if these measures are carried out in urban areas.
- In ambient noise measurements of buildings, in which their dynamic characteristics, such as their natural frequencies and mode shapes, may be evaluated [18].

Furthermore, the use of seismic array methods for estimating the landslide structure and the thickness of materials [22] within the framework of a research project (CGL2015-65602-R), currently in progress, has shown the difficulties of conducting field work using devices connected by cables, especially when the arrays are large, and there is vegetation in the area.

Therefore, the wireless management of the arrays would have great advantages in this kind of measurement. In this sense, the developed system consists of a central control unit, which manages all the communications with the remote stations wirelessly. A laptop is connected with the central unit in order to provide a graphical interface to the user, who can insert the different configuration parameters and follow the progress of the entire acquisition process.

Finally, another important issue related to the array measurements is the position of the stations. Their location has to be accurately determined in order to apply any of the methods mentioned in the previous section. In general, the inter-station distances are obtained in the field using a measuring tape, but sometimes this is not easy, especially for large arrays or in urban environments, where there can be obstacles to distance measurements. In this sense, the developed system incorporates a GPS module in each of the remote stations, which provides the position of each one. Thus, we avoid measuring the inter-station distances in the field, saving time in the array deployment and allowing for the implementation of any arbitrary geometry.

### 2.2. System Hardware

The developed system, Geophonino-W, constitutes a piece of completed wireless seismic recorder equipment. It is based on the Arduino Due board, whose general specifications are shown in Table 1, and a set of several Arduino shields.

The Geophonino-W system basically consists of two different types of modules: the Management Control Node (MCN), controlled by a laptop; and the Acquisition Control Node (ACN), connected directly to the sensor and remotely controlled by the MCN. One MCN is able to manage between one to twelve ACNs, each of which can also record from one to twelve channels simultaneously, although in our case, we use it to register only 1 or 3 channels, depending on whether they are used with vertical or triaxial sensors.

**Figure 1:**
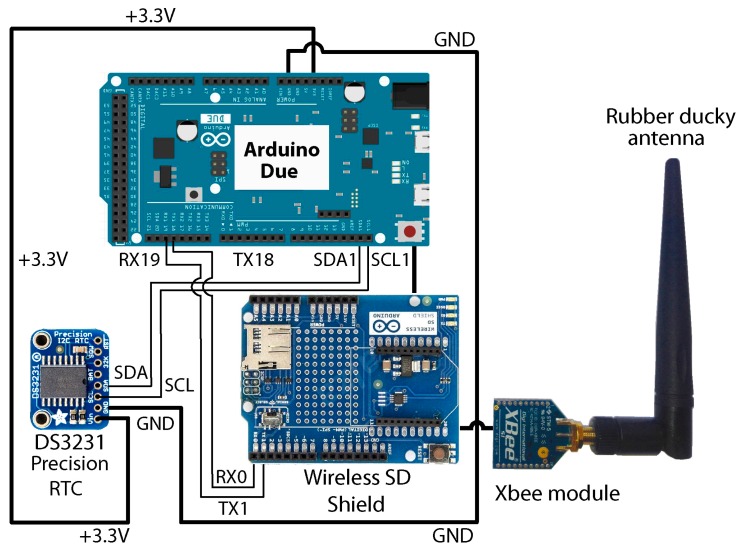
Diagram of the components and connections of the Management Control Node (MCN) system.

The MCN consists of five components (Figure 1):

- An Arduino Due board;
- A DS3231 Precision RTC (Real time clock), with a 3 V CR1220 40 mAh battery;
- An Arduino Wireless SD Shield, with an 8 Gb SD card;
- An Xbee S1 802.15.4 low-power module, with an RPSMA connector;
- A 2.4 GHz omnidirectional rubber ducky antenna, model HG2403RD, with a gain of 3 dBi (decibels relative to the isotropic).

**Figure 2:**
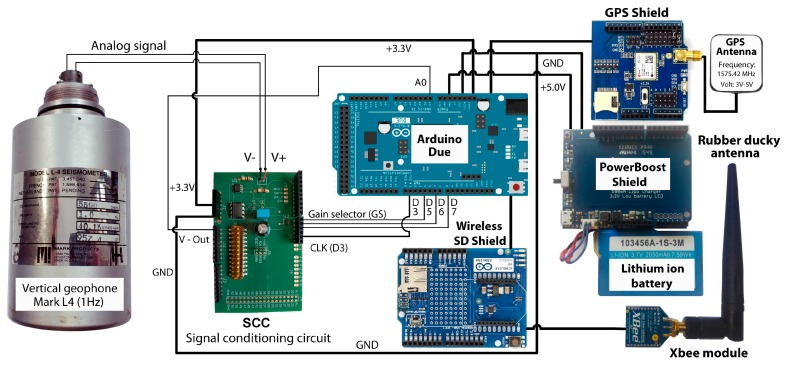
Diagram of the components and connections of the Acquisition Control Node (ACN) system.

Meanwhile, the ACN module (Figure 2) consists of the following elements:

- An Arduino Due board;
- An Arduino Wireless SD Shield, with an 8 Gb SD card;
- An Xbee S1 802.15.4 low-power module, with an RPSMA connector;
- A 2.4 GHz omnidirectional rubber ducky antenna, model HG2403RD, with a gain of 3 dBi (decibels relative to isotropic);
- An ITEAD RoyalTek REB-5216 GPS Shield Breakout Board;
- A GPS active antenna, model IM120717003;
- A PowerBoost shield adapter, with an output of 5 V 500 mA;
- A Lithium ion 3.7 V 2050 mAh rechargeable battery, model 103456A-1S-3M;
- A Signal Conditioning Circuit (SCC).

**Figure 3:**
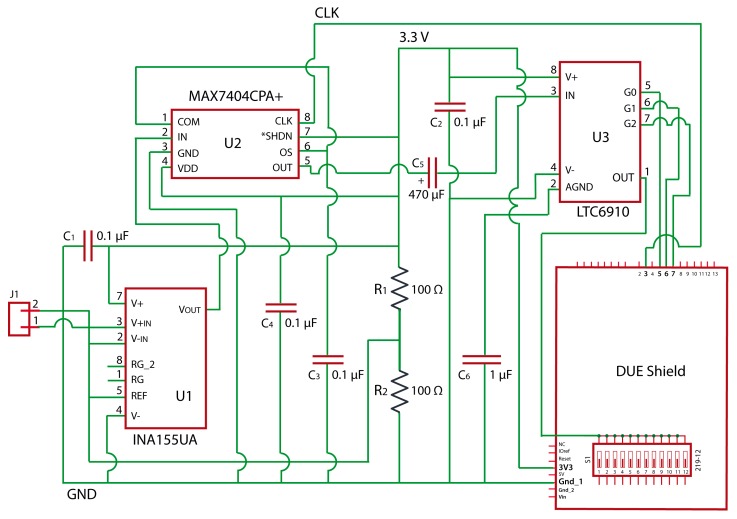
Schematic circuit of the Signal Conditioning Circuit (SCC) (made with Eagle free software).

**Figure 4:**
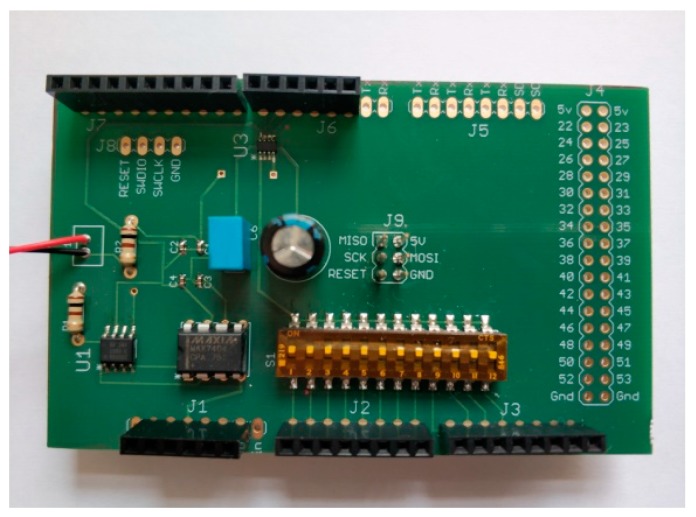
Example of an SCC with welded components.

A schematic circuit of the SCC is shown in Figure 3. It includes three main components: An instrumentation amplifier, INA155; an anti-aliasing filter, MAX7404, which is an 8th-order, low-pass, elliptic, switched-capacitor filter; and a programmable gain amplifier, LTB5 86910-1. The capacity of this design for the conditioning of seismic signals has been demonstrated in reference [23]. In order to physically implement the circuit, a PCB has been designed (Figure 4). The PCB design scheme can be downloaded from https://github.com/JLSolerLlorens/Geophonino-W.

**Figure 5:**
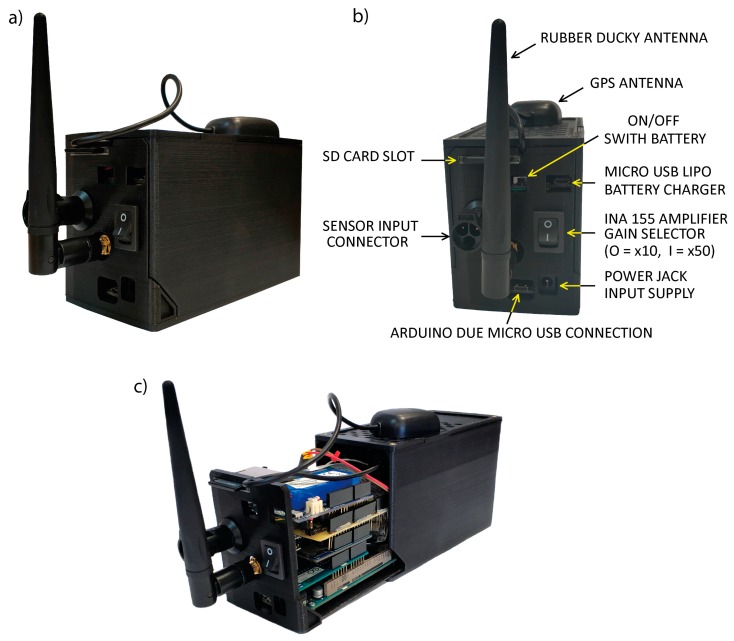
(**a**) Closed ACN enclosure. (**b**) ACN enclosure with indications about input connections and switches. (**c**) ACN component distribution inside the enclosure.

All ACN and MCN components were integrated in a casing (Figure 5). They were designed using Blender software and can be printed using a basic 3D printer. The files corresponding to the ACN and MCN housing modules can be downloaded from: https://github.com/JLSolerLlorens/Geophonino-W. Our design is a modification of the original design, published by Paul Homes [24] under a Creative Commons Attribution-Non-Commercial license. The total cost of all the components of an ACN single-channel recorder system is around 290 €, and the cost of all of the components of an MCN is approximately 100 €.

### 2.3. System Communications

Two standard specifications might be used to implement the developed system, ZigBee and Bluetooth. The Bluetooth protocol presents a higher transmission rate than Zigbee (3 Mbps versus 250 kbps) and a greater immunity to noise and interferences. However, for our application purposes, the Zigbee specification results are more suitable. It allows for the configuration of a low-power, low-complexity, low-cost, wireless mesh network, which is appropriate for field campaigns. Besides, it allows the maximum transmission distance to be much larger (100 m) than that provided by the Bluetooth technology (10 m) [25], which is a crucial factor for seismic noise array deployment. 

The Zigbee protocol is implemented in Xbee modules (Digi International Inc. Hopkins, MN, United States), which constitute the core of developed communication systems. These modules are based on the IEEE 802.15.4 specification and work in the frequency band of 2.4 GHz.

The Xbee S1 802.15.4 low-power module with an RPSMA connector has been selected from the existing range of Xbee modules, since it is cheaper than the other Xbee models. Its RF line-of-sight ranges are 90 and 30 meters in outdoor and indoor/urban areas, respectively, which is enough for most array configurations. For example, for a circular layout, with the MCN located in the center, the maximum aperture might reach 180 m. In any case, if a larger aperture were required, the Xbee S1 modules could be replaced by the Xbee-Pro ones, which allow for line-of-sight communications of 1600 and 750 m in outdoor and indoor/urban areas [26], respectively, with slight modifications to the software.

Xbee modules can be configured in Transparent (AT) or in Application Programming Interface (API) modes. By default, they are in the AT mode. However, in this work, the API mode has been selected, as this allows for the identification of the origin and destination of all of the data packets and can be controlled by the microcontroller. In order to simplify the management of the data packets, we have used the XBee-Arduino General Public License (GPL) library, developed by Andrew Rapp [27].

The configuration of the Xbee modules is achieved through the XCTU software (Digi International Inc.) and involves the following steps:

(1) Selection of the API mode. In the serial interfacing options, the AP parameter is changed to “AP-enabled w/PPP”.
(2) Definition of a common network. In order to allow for communication among all the Xbee modules, they have to be included in the same network. To achieve this, the Pan ID parameter is set up with the same value for all nodes. In our case, the 855 value has been selected.
(3) Definition of the ACN and MCN addresses. A 16-bit source parameter has to be defined for each component of the wireless network. In our case, the ACN devices have been numbered from 1 to 12, and the MCN module has been labeled with the number 13. By default, the system has been designed for a maximum of 12 ACN devices, but it can easily be modified, with minor changes to the Arduino Due code.

Once the configuration process is complete, the XCTU software allows the configuration profile in the internal memory of each Xbee module to be saved. Therefore, this process is only required once, before the first use of the system, since the configuration parameters will remain recorded in the memory. An example of a configuration file can be downloaded from https://github.com/JLSolerLlorens/Geophonino-W. 

**Figure 6:**
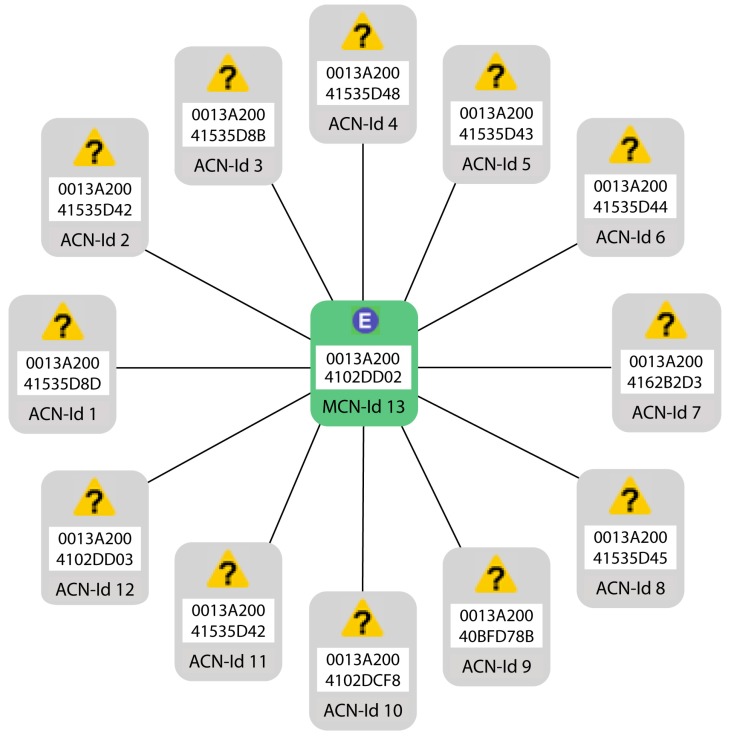
Overview of all of the Xbee modules connected in the star topology.

In Figure 6, an overview of all of the devices connected in the star topology is shown. This view is obtained from the XCTU software.

### 2.4. Design and Implementation of the System Software 

Three different programs have been developed for the implementation of the Geophonino-W system: two for the functioning of the MCN and ACN modules (developed in the Arduino programming’s language); and another one for the Graphical User Interface (GUI) (implemented by Processing, an open-source language). In the next sections, the software implementation for each module and for the GUI is described in detail. 

#### 2.4.1. MCN Software Implementation

**Figure 7:**
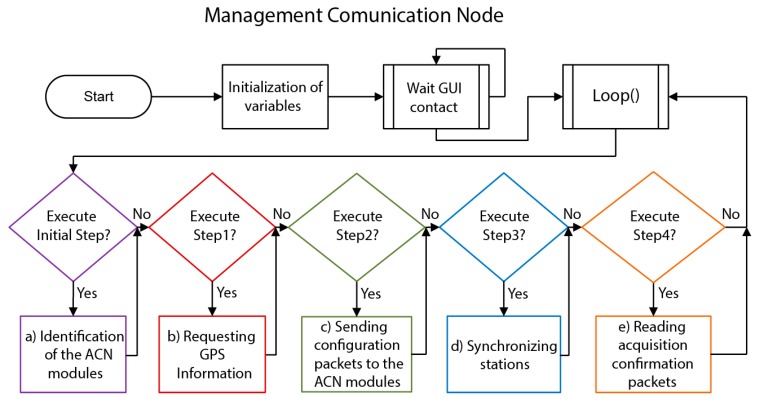
General flowchart of the MCN software.

**Figure 8:**
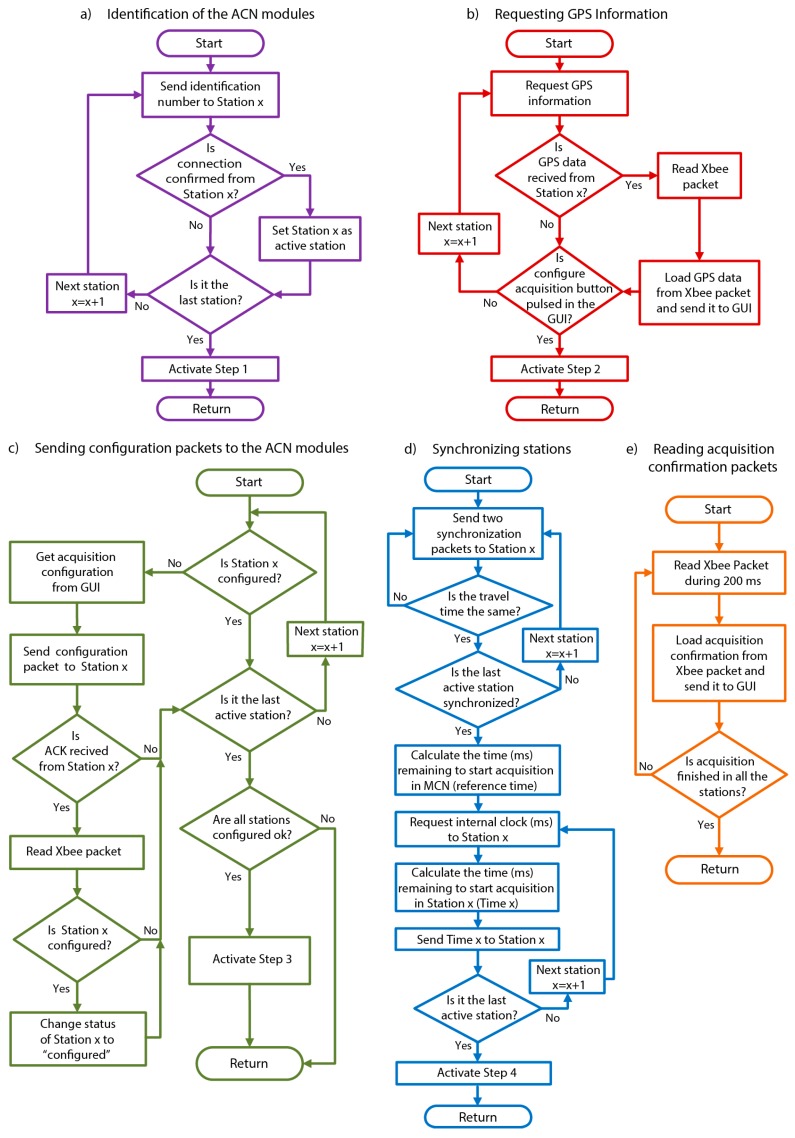
Detailed flowchart of the MCN software.

In Figure 7 and Figure 8, the general structure and a detailed flow chart of the MCN software are shown. Once the system is switched on, the different system variables are declared and initialized. After that, the system remains in standby, waiting for a GUI message through the serial port. When the GUI sends a connection message to the MCN, the program enters into a loop section, carrying out the following processes sequentially:

1. Identifying the ACN modules within the array
  In the first step, the MCN assigns a different identification number to each one of the ACN modules integrated in the array of sensors. This identification number is sent to each of the ACN modules and used subsequently in all the communications between the MCN and the ACNs.
2. Requesting GPS information from all the ACN modules
  If any of the stations did not respond at least once to the requests, it is discarded for the rest of the operations.
3. Sending configuration packets to the ACN modules
  The MCN sends the configuration packets to the different ACN modules. The correct sending of data is controlled through an error communication counter (ECC), which the MCN module activates for each ACN at the beginning of the process. In this way, if the MCN module did not receive any response from any of the ACN modules after 40 attempts (i.e., the ECC reaches the value of 40), then those ACNs would be marked as inactive stations and would not receive further communication attempts from the MCN. The data validation is conducted in the respective ACN modules, which respond to the MCN with a confirmation packet, if everything is correct. If any error was detected, the ACN would respond with an error packet, and the MCN would re-send the configuration packet.
4. Synchronizing the internal clock of each ACN module with the MCN clock
  Array measurements require that all of the stations in the array register simultaneously, so a precise synchronization is required between all the stations [11]. Thus, one of the most important tasks of the MCN is to ensure the strict synchronization between ACNs. The implemented synchronization process is carried out in three main steps. Firstly, the MCN sends a message to each of the ACN modules and waits to receive the answer from the corresponding ACN, measuring the associated travel time. This is repeated twice. The aim is to ensure that the travel times are the same for all of the ACN modules. In the second step, the MCN module calculates the time, in milliseconds, that remains to start the data acquisition. This time is taken as a reference time and should be at least longer than 20 seconds, as this is approximately the time required to synchronize the 12 stations used in this work. Finally, the MCN module asks each ACN for the value of its internal clock, in milliseconds. In this way, taking as the reference time the one calculated in the second step, the time at which the data acquisition should start in each of the ACN modules is obtained and sent to each of them.
5. Reading acquisition confirmation packets from the ACN modules
  During the data acquisition process, each one of the ACN modules sends an acquisition confirmation packet to the MCN every 3 seconds. In this way, if any problem occurred in any of the remote stations (e.g., low battery), then the MCN would detect the ACN that fails. Anyway, the data acquisition would continue with the available active ACN modules.

#### 2.4.2. ACN Software Implementation

**Figure 9:**
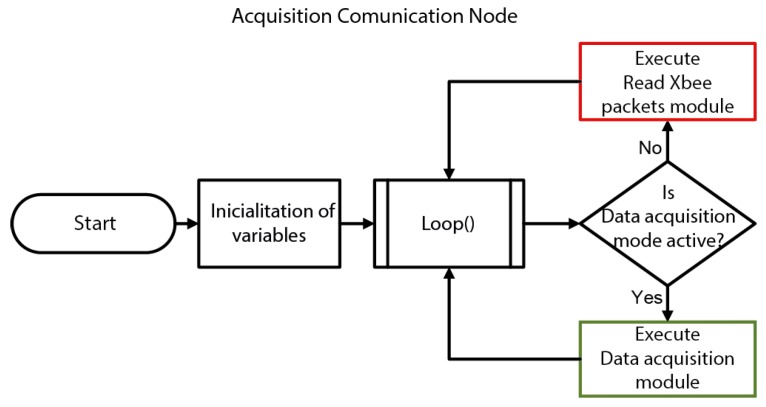
General flowchart of the ACN software.

In Figure 9, a general flow chart of the ACN program is shown. Once the system is switched on, it starts declaring and initializing the required variables. The ACN has two principal modes of operation, depending on whether the acquisition mode is enabled or not.

**Figure 10:**
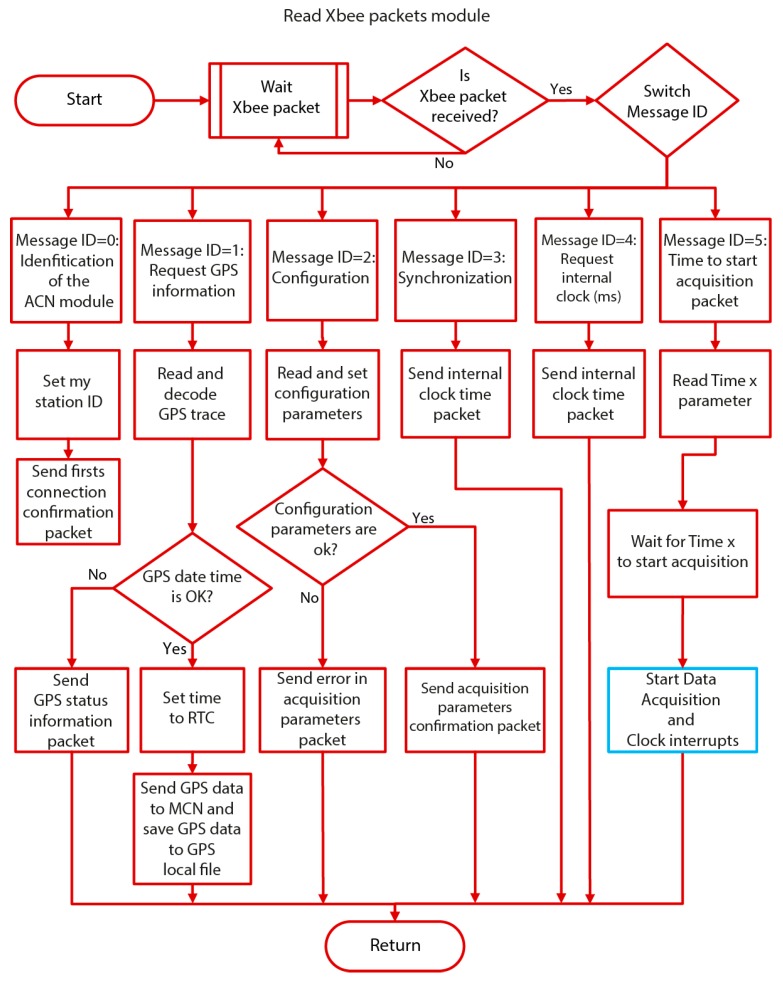
Detailed flowchart of the ACN software (“Read Xbee packets module”).

**Figure 11:**
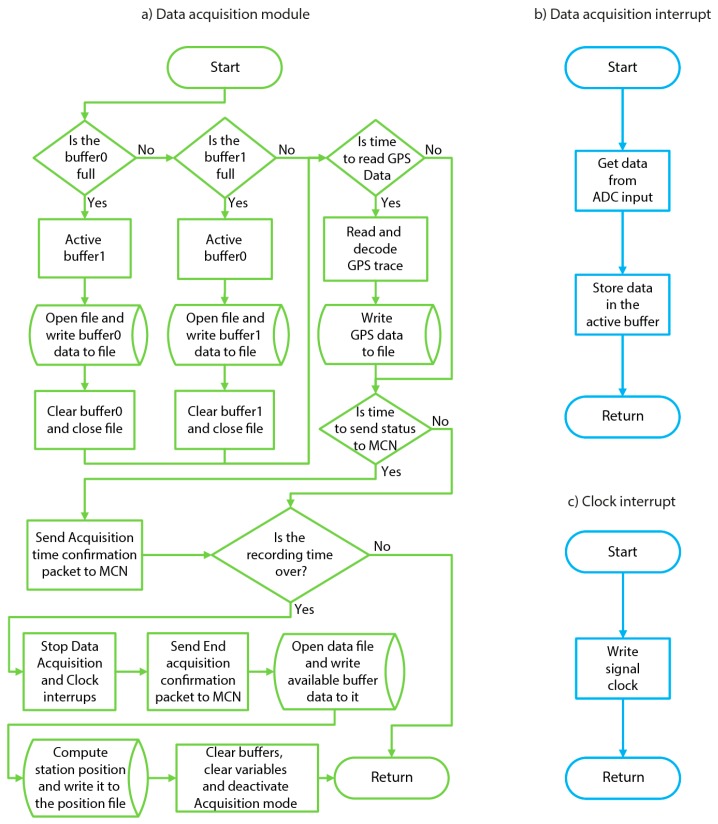
Detailed flowchart of the ACN software (“Data acquisition module”) and the associated interrupts.

When the acquisition mode is not enabled, the ACN continues to wait for an Xbee packet from the MCN (“Read Xbee packets” module). In Figure 10, a detailed flowchart of the program associated with this module is shown. In this stage of the process, the ACN acts according to the type of message received from the MCN. The structure of these messages (i.e., the Xbee packets) is {Message ID, Station ID} or {Message ID, Station ID, data}. The Message ID indicates the function or process to carry out, and the Station ID indicates the number of the station associated with the corresponding ACN. Some of the functions used require the sending of some additional data. In this case, they are appended after the Station ID parameter. The different processes, related to those of the MCN software (Section 2.4.1), are:

1. Identification of the array stations: {Message ID = 00, Station ID = 01, 02 … 12}
  All of the ACN modules are programmed identically. Therefore, in the first step, each of the ACN modules has to be identified within the array. To achieve this, the MCN assigns a different Station ID for each of the ACN modules (i.e., Station ID = 01, 02 … 12) and sends the corresponding identification to each of them. From now on, the Station ID parameter is included in all the messages between the MCN and ACN for identification purposes.
2. Sending GPS information to the MCN module: {Message ID = 01, Station ID = 01, 02 … 12}
  The MCN module uses this Message ID to request GPS information from each of the ACN modules. When one ACN receives this Xbee packet, the program reads and decodes an NMEA GPS trace. The management of the NMEA GPS data packets has been carried out using the TinyGPS (GPL) library [28].
  Once the GPS information is obtained correctly, the ACN sets its internal RTC with the obtained date and time. Besides, the GPS data are sent to the MCN via the Xbee connection and saved in the SD memory card of the corresponding ACN. The GPS information is saved in an ASCII file, whose name is composed of the time and the date, and the extension is formed by the letter ‘g’, plus the Station ID (e.g., ‘140512_05022019.g01’). This file contains two header lines. The first line contains the Station ID, and the second line contains the description of the saved parameters, that is: Time, Latitude, Longitude, HDOP (Horizontal Dilution Of Precision), and NumSatellites. The HDOP information includes the Horizontal Dilution Of Precision, and NumSatellites includes the number of satellites used to determine the position. This information is provided for each GPS trace.
3. Receiving and saving configuration data: {Message ID = 02, Station ID = 01, 02 … 12, Data}
  This Xbee packet contains all the configuration parameters required for the data acquisition. When this packet is received, the ACN system extracts the different configuration values and saves them in the corresponding local variables of the program. After that, the ACN module sends an Xbee packet to the MCN, indicating whether all of the configuration parameters are correct (confirmation packet) or not (error packet).
4. Sending the synchronization packets to the MCN: {Message ID = 03, Station ID = 01, 02 … 12}
  When any of the ACN modules receives an Xbee packet with a Message ID equaling 3, the associated ACN automatically returns a confirmation packet to the MCN, which is in charge of measuring the travel time between MCN and ACN. This process is repeated twice for each of the ACN modules in order to verify that the travel times are the same for all of them.
5. Sending the internal clock to the MCN: {Message ID = 04, Station ID = 01, 02 … 12}
  When any of the ACN modules receives a Message ID equaling 4, the associated ACN automatically returns its internal clock (in milliseconds) to the MCN, which estimates the time at which the data acquisition should start in that ACN module.
6. Starting data acquisition in the millisecond: {Message ID = 05, Station ID = 01, 02 … 12, T}
  This Xbee packet contains the time (in milliseconds) that it has to reach the associated ACN module, before starting the data acquisition (“Data acquisition” module). During the data acquisition, two interrupts are used. The Data Acquisition Interrupt process uses an Interrupt Service Routine (ISR), which ensures that the samples are acquired at the precise sampling period selected by the user. The Clock Interrupt routine is used to send a signal clock to the MAX7404 (see Section 2.2), which establishes the cut-off frequency of the anti-aliasing filter. In the proposed system, the available sampling frequencies are 100 Hz, 250 Hz and 500 Hz, and the corresponding cut off frequencies are 40 Hz, 100 Hz and 200 Hz, respectively. In Figure 11, a detailed flowchart of the program associated with the “Data acquisition” module, as well as the two interrupts used, is shown.

Once starting the data acquisition, each of the ACN modules temporally stores the data in two different buffers, alternately, in order to avoid possible overflow problems. During this stage, these additional functions are carried out by the ACN modules:

- Dumping the memory buffers on the SD card. When either of the two buffers are full, the other buffer is automatically activated, and the data are saved in the SD card. The data file is opened and closed in each buffer dump operation. In this way, in case of an unexpected stop of the data acquisition, all of the data are saved, until the moment of the unexpected stop. This is an important improvement in relation to other previous implementations [23,29], where the data file was opened and closed just once, at the beginning and the end of the data acquisition, respectively.
- Reading and decoding the NMEA data and saving the GPS information in a data file. In order to obtain a more reliable estimation of the position, GPS information is taken every three seconds and appended to the ASCII file, created in step 2 ({Message ID = 01}). The interval time for this operation might be easily changed, but our experiments prove that a three-second interval is enough to estimate the correct position of each station within the array.
- Sending a confirmation Xbee packet to the MCN. Each of the ACN modules sends a message every three seconds to the MCN in order to confirm that the data acquisition is being done correctly.
- Checking the end of the data acquisition. When the acquisition time is completed, the interrupts are stopped, the buffers are dumped on the SD card and an end-of-acquisition Xbee packet is sent to the MCN module. After that, the station position is computed, all acquisition variables are cleared, and the system returns to the “1. Read Xbee packets module” mode. In this mode, the system continues to wait for new Xbee packets in order to start and complete the described process all over again.

As for the station position, estimated at the end of the data acquisition, this is calculated as the weighted arithmetic mean of all of the GPS positions obtained during the acquisition process (Equation (1)).

(1) $$X_{mean}=\frac{\frac{1}{HDOP_{1}}\;X_{1}+\frac{1}{HDOP_{2}}\;X_{2}+\ldots +\frac{1}{HDOP_{n}}\;X_{n}}{\frac{1}{HDOP_{1}}+\frac{1}{HDOP_{2}}+\ldots +\frac{1}{HDOP_{n}}}$$

where X is the latitude/longitude, and the inverse of the HDOP value is the respective weight. The HDOP value is directly proportional to the position error. Therefore, a low HDOP value means a minor error in the obtained position. Meanwhile, a high HDOP value implies a greater error in the position. The use of the weighted mean value, instead of a single GPS measure, provides a much more accurate estimate of the position of each one of the stations, which is of great importance for the correct subsequent analysis of the data recorded by the array.

The complete data acquisition returns three different data files, saved in the corresponding SD cards of each one of the ACN modules. The name of these three files consists of the time and date of the start of the acquisition. The data information (time and amplitude) is saved with the extension, ‘s’, plus the Station ID (e.g., ‘140512_05022019.s01’). The GPS positions collected during the data acquisition are saved with the extension, ‘g’, plus the Station ID (e.g., ‘140512_05022019.g01’). Finally, the weighted arithmetic mean of the GPS positions is saved with the extension, ‘p’, plus the Station ID (e.g., ‘140512_05022019.p01’).

#### 2.4.3. Graphical User Interface

**Figure 12:**
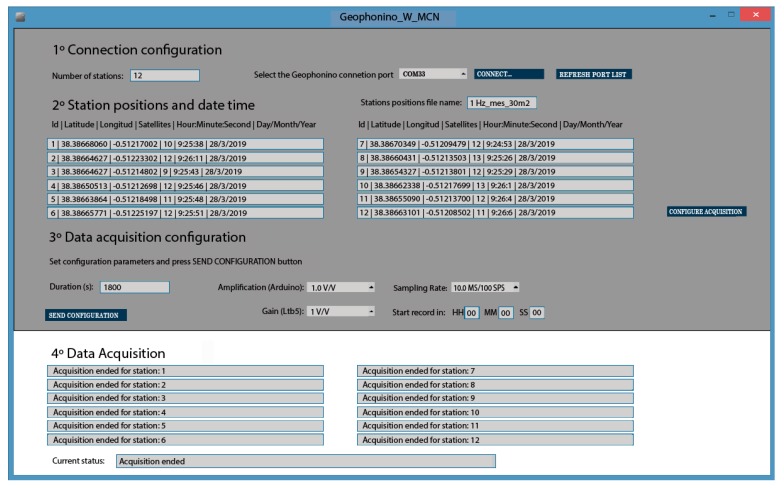
Graphical User Interface (GUI) of Geophonino-W. Screenshot after finishing a data acquisition.

The GUI has been developed with Processing, an open-source programming tool that is compatible with the main operating systems (e.g., Mac OS X, Windows, Linux or Android). The GUI guides the user step-by-step through the entire configuration process (Figure 12). Four main steps establish the configuration and the data acquisition processes.

1. Connection configuration. In this step, the user selects the number of remote stations and the serial port, where the MCN is connected.
2. Station positions, date and time. In this part of the process, the MCN receives the GPS information corresponding to each one of the ACN modules and sends the respective position, date and time to the GUI. A station position file’s name is also defined in this step. This file is saved in the SD card of the MCN module and contains one line for each ACN station, with the following parameters: ACN identification, latitude, longitude, number of satellites, hour and date. When the file name is introduced, and the GPS data of all the stations are received correctly, the button, “Configure acquisition”, is activated, allowing us to progress to the next step.
3. Data acquisition configuration. The parameters required for the data acquisition are introduced in this step. These parameters are: recording duration (in seconds); Arduino Due amplification (1, 2, 4); LTB5 amplification (0, 1, 2, 5, 10, 20, 50 and 100); sampling rate (100, 250 and 500 Hz) and the time remaining to start the acquisition (hour, minute and seconds). When the *Send Configuration* button is pressed, all these parameters are appended at the end of the station position file, created previously in step 2.
4. Data acquisition. In this last step, the MCN module accomplishes processes 3, 4 and 5 (see Section 2.4.1) and sends information about the progress of these processes to the GUI. In this way, different messages are appearing progressively in the GUI, which are related to the configuration, synchronization and data acquisition for each one of the stations.

## 3. System Characterization and Tests

The performance of the developed Geophonino-W system has been accomplished through several laboratory experiments. The tests carried out are related to the frequency response, precision, accuracy and battery duration of each one of the ACN modules. Besides, the synchronization of the data recorded by all of the ACN modules of the system has been also assessed.

### 3.1. Frequency Response

**Figure 13:**
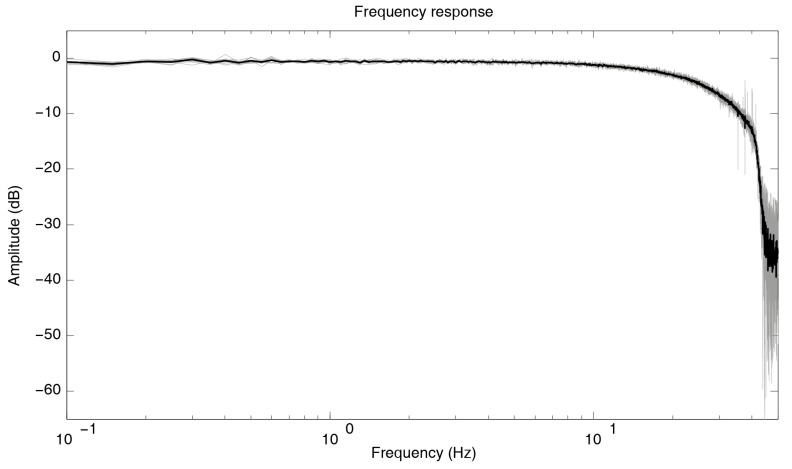
Frequency response of the 12 analyzed ACN modules (light gray), together with the average frequency response (black line).

In this first analysis, the transfer function of each one of the ACN modules has been obtained experimentally. The main objective is not only to know the frequency response of the system, but also to demonstrate the repeatability of the results for each one of the twelve stations used. To achieve this, an impulse signal (provided by a Tektronix AFG310 function generator) was applied simultaneously to the twelve ACN modules, obtaining the impulse response function and, subsequently, the frequency response for each system. For this experiment, 20 seconds were recorded, with a sampling frequency of 100 Hz. In Figure 13, the frequency response of all of the analyzed ACN modules is shown, together with the average result. The consistency between all the ACN modules is clearly observed in this figure. The range of the Geophonino-W is established by the MAX7404 low-pass filter, which is used as an antialiasing filter. The theoretical cut-off frequency of this filter is 40 Hz, but in practice, the decay of the signal can be observed from approximately 30 Hz. Anyway, it does not have an influence on the passive survey, because the frequency range of interest for site effect estimation purposes is lower than 10 Hz [30], and the maximum frequency of engineering interest is 20 Hz [31].

### 3.2. Precision and Accuracy Tests

**Figure 14:**
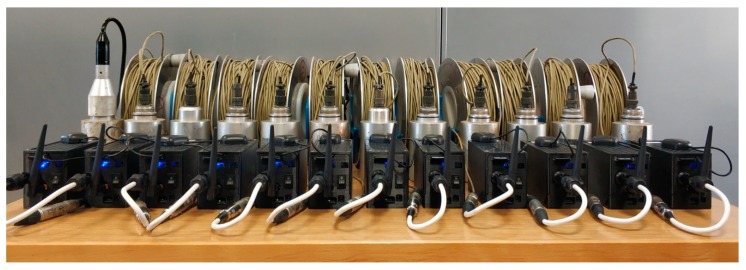
ACN modules connected to Mark L4 1 Hz sensors.

In order to study the precision and accuracy of the developed acquisition systems, two different experiments were carried out in the laboratory, with ambient noise measurements. In all cases, the equipment used was placed on a table in the laboratory (e.g., Figure 14), and 30 minutes were recorded, with a sampling frequency of 100 Hz. 

#### 3.2.1. Precision

**Figure 15:**
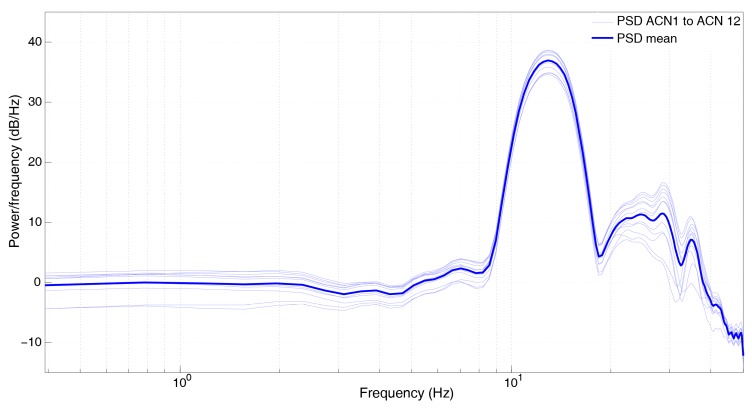
Power Spectra Density of the normalized recordings of 12 ACN modules, connected to 12 Mark L4 1 Hz sensors.

In this experiment, each of the 12 ACN modules was connected to a different Mark L4 1 Hz sensor, registering all of them simultaneously (Figure 14). In Figure 15, we show the Power Spectra Density (PSD) of the normalized recordings, together with the average curve (thick line). A great repeatability is observed among all of the sensor–ACN pairs analyzed. 

#### 3.2.2. Accuracy

**Figure 16:**
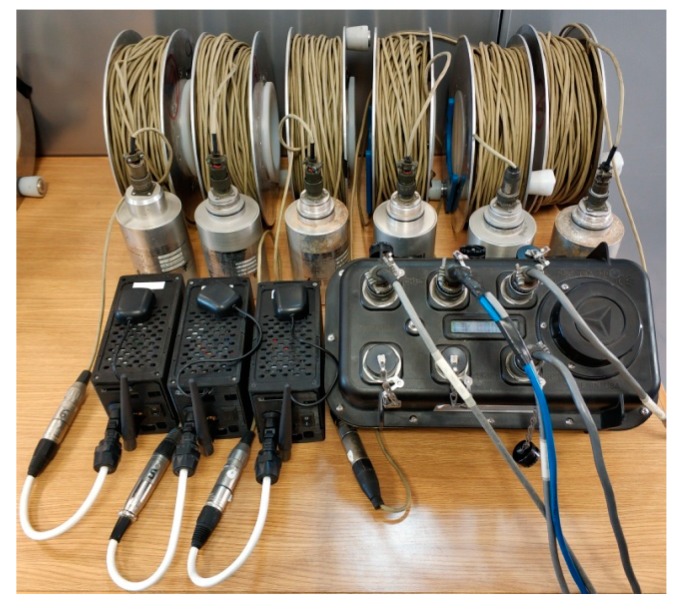
Mark L-4 sensors connected to the commercial equipment, Reftek, and the developed acquisition system, Geophonino-W.

**Figure 17:**
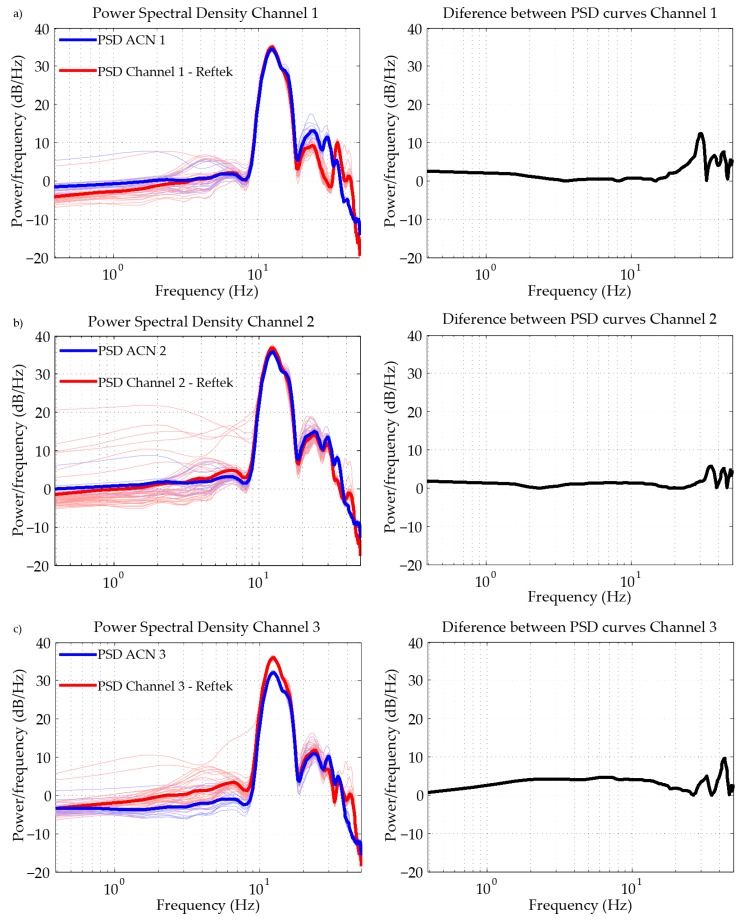
Power Spectra Density of the normalized recordings of 3 ACN modules (blue lines) and the three channels of the commercial equipment, Reftek (red lines). (**a**) Vertical, (**b**) North-South and (**c**) East-West component. The thick lines correspond to the average curves. In the right column, the absolute differences between the average PSDs of each system are shown.

In this test, six Mark L4 1 Hz sensors were used, three of which were connected to three ACN modules, with the other three connected to the three channels of a commercial recorder system, Reftek (Figure 16). The equipment started to register inputs from all sensors almost simultaneously. In the left part of Figure 17, we show the PSD of the normalized recordings from the ACN modules (blue lines) and each of the channels of the Reftek (red lines) for all of the analyzed time windows. The average PSDs are also shown in these plots with a thick blue/red line. In the right part of Figure 17, the absolute differences between the average PSDs of each system are shown. The correlation coefficients between the average PSDs are 92, 98 and 96%, and the average difference values are 3.7, 2.0 and 3.3 dB/Hz for channels 1, 2 and 3, respectively. In the Reftek system, channels 1, 2 and 3 correspond to the Vertical, North-South and East-West components, respectively. The comparison between the average PSD curves reflects a good agreement in the complete frequency band, which indicates of a good accuracy, relative to the commercial equipment used.

### 3.3. Synchronization Test

**Figure 18:**
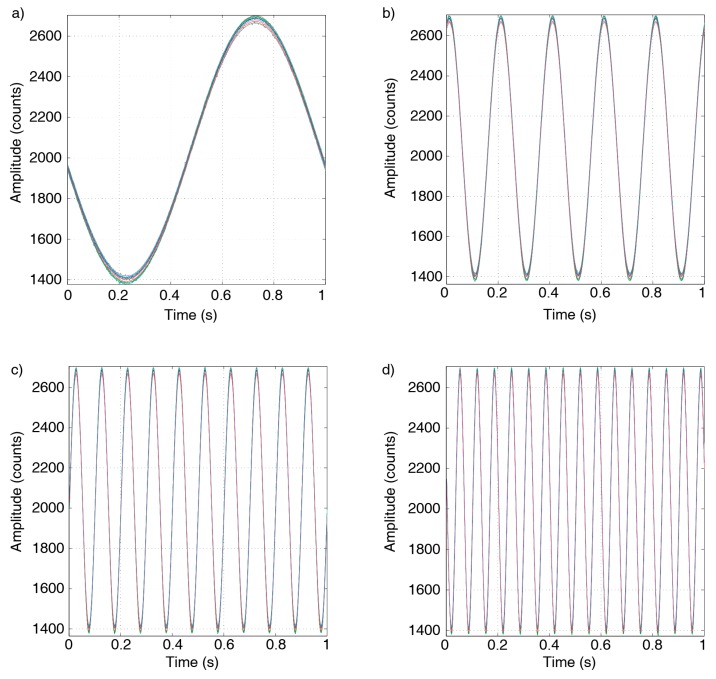
Synchronization test for sine waves in the range of 1–15 Hz, with 0.1 V amplitude. (**a**) 1 Hz; (**b**) 5 Hz; (**c**) 10 Hz; (**d**) 15 Hz. Twelve simultaneously registered signals are shown in each test.

The next laboratory experiment focused on the analysis of the strict synchronization of the ACN modules. In order to analyze the correct system synchronization, a common input signal was connected simultaneously to the 12 ACN modules, and then the corresponding recorded signals were compared. Several 0.1V input signals were used for this test, from 1 to 15 Hz, using the Tektronix AFG310 function generator. The ACN modules were configured with gains of 20 dB (×10), 0 dB (x1) and 0 dB (x1) for the INA155, LTB5 and Arduino Due amplifiers, respectively; and the selected sampling frequency was 500 Hz. The recording duration was set to 10 s. In Figure 18, we show the first second recorded by the 12 ACN modules for the frequencies of 1, 5, 10 and 15 Hz. The obtained results clearly show a good synchronization among all the stations for the frequency range of interest.

### 3.4. Battery Duration Test

Each of the ACN modules is equipped with a lithium ion 3.7 V 2050 mAh battery for autonomous registration (see Section 2.2). Therefore, the maximum registration time is limited by this battery. In this test, we left the 12 ACN continuously registering modules in order to estimate the maximum time of autonomous acquisition. In this case, the average duration of the 12 ACN modules was 274 ± 10 min. This duration might be increased simply by using a lithium ion battery of a higher capacity. The consumption of the system modules was measured with an ammeter, and it was 205 and 290 mA for the MCN and ACN, respectively.

Alternatively, an external battery might be used. Thus, we tested the use of 4 batteries of 1.5 V AA, which are very common batteries and can be bought almost anywhere. In this case, the estimated duration was 447 ± 10 min.

## 4. Field Test for Validation

Seismic methods, mentioned in the introduction, include active and passive methods. The main difference between them is the source signal required for data acquisition. Active sources, such as a weight drop, explosives or seismic vibrator vehicle, are used in active methods, and passive sources, such as seismic noise, are used in passive methods. In general, active methods use information on high frequencies, offering a good resolution but a lower penetration. Meanwhile, passive methods use data on low frequencies, allowing them to arrive at greater depths. Hence, passive methods are based on the measurement of the ambient noise produced by natural phenomena or by human activities, avoiding the requirement of an artificial seismic source, which is essential for active seismic methods. Seismic noise signals are composed by body and surface waves, although the latter are the ones that contribute the most to the total amount of energy [32] and are the most coherent components in the signal [33]. Thus, an analysis of the surface waves allows us to obtain the soil characteristics of the site under study. Passive methods are increasingly used to study soil characteristics and associated site responses, because they are less expensive than geotechnical methods, and they are also non-invasive, which is highly recommended for urban areas [30].

A wide variety of passive techniques have been developed and applied in the last 70 years to analyze seismic noise. Most of them are based on array measurements, in which the analysis of the signals, recorded simultaneously by a set of sensors, provides information about the propagation characteristics of surface waves (velocity-frequency response or dispersion curve) in analyzed soil. Thus, the dispersion curve constitutes one of the parameters used to characterize soil. Some of the most widely used analysis techniques are the multichannel analysis of the surface wave (MASW) [34] method, the refraction microtremor (ReMi) [35] technique, the spatial autocorrelation (SPAC) [36] analysis, the extended spatial autocorrelation (ESAC) [37] method and the frequency-wavenumber (f-k) [38] transform. These array methods are often used for soil characterization purposes in urban areas [39]. The inversion of the dispersion curve [40,41,42,43,44] allows the shear-wave velocity profile, i.e., the S-wave velocity and the thickness of the different layers that characterize the soil under study, to be estimated. A complete revision of the array methods and their applications can be found in reference [4]. 

One of the most widely used array techniques is the f-k method [33,38,45], and it is the method that we have applied in this work. The f-k method is based on the assumption that a spectral density function can characterize a stationary random process. In the same way, a frequency-wavenumber spectral density function could characterize seismic noise [39]. 

In order to evaluate the performance of the developed Geophonino-W system in seismic noise array measurements, different field microtremor surveys were carried out at four sites around the province of Alicante (Southeastern Spain). Concretely, the selected sites were Almoradí (−0.78292º, 38.11184º), Rojales (−0.72022º, 38.09128º), Catral (−0.836197º, 38.147742º) and Mutxamel (−0.446117º, 38.424331º). In each of the sites, a circular-shaped array was deployed with 1 Hz vertical sensors (Mark L4). The data acquisition was configured with the following characteristics: A sampling frequency of 100 Hz, recording time of 30 minutes, Arduino Due gain of 0 dB (×1) and INA155 gain of 20 dB (×10). In the case of the LTB-5 amplifier, different gains were tested in order to study the behavior of the system at different sites and with different noise characteristics. As a result, we finally selected the voltage gain of 34 dB (×50) for Almoradí and Rojales, 26 dB (×20) for Mutxamel and 0 dB (×1) for Catral. The study suggests the use of amplifications below 34 dB (×50), as higher amplifications might saturate the signal at some sites.

At these selected sites, array measurements had previously been taken by us for soil characterization and array measurement optimization purposes. Concretely, they were carried out in 2007 for Mutxamel [46], in 2011 [39] for Rojales and Almoradí, and in 2014 [47] for Catral. An Earth Data pr6-24 [48] portable model 24-bit digitizer, connected to nine Mark L4 sensors, was used for the data acquisition in Rojales and Almoradí. The same digitizer, but with only five Mark L4 sensors, was used in Mutxamel. In the work developed in Catral, a RAS-24 exploration Seismograph [49], connected to nine 10 Hz vertical geophones, was employed. Thus, the comparison of the Rayleigh wave dispersion curves obtained using the developed system and the ones obtained using these commercial digitizers might provide a good way to determine the reliability of the proposed Geophonino-W system.

This comparison is possible, because the used technique is based on the assumption of a stochastic wavefield, which is stationary, both in time and space [36]. In one related study, developed by Endrun et al. [50], the temporal consistency and repeatability of the array analyses is evaluated. The results obtained by them show a good correlation in the obtained dispersion and autocorrelation curves for two different field campaigns. In a similar way, Rosa-Cintas et al. [51] analyzed the consistency of two array measurements, carried out in different years and with different kinds of stations in the area under study in the present paper.

**Figure 19:**
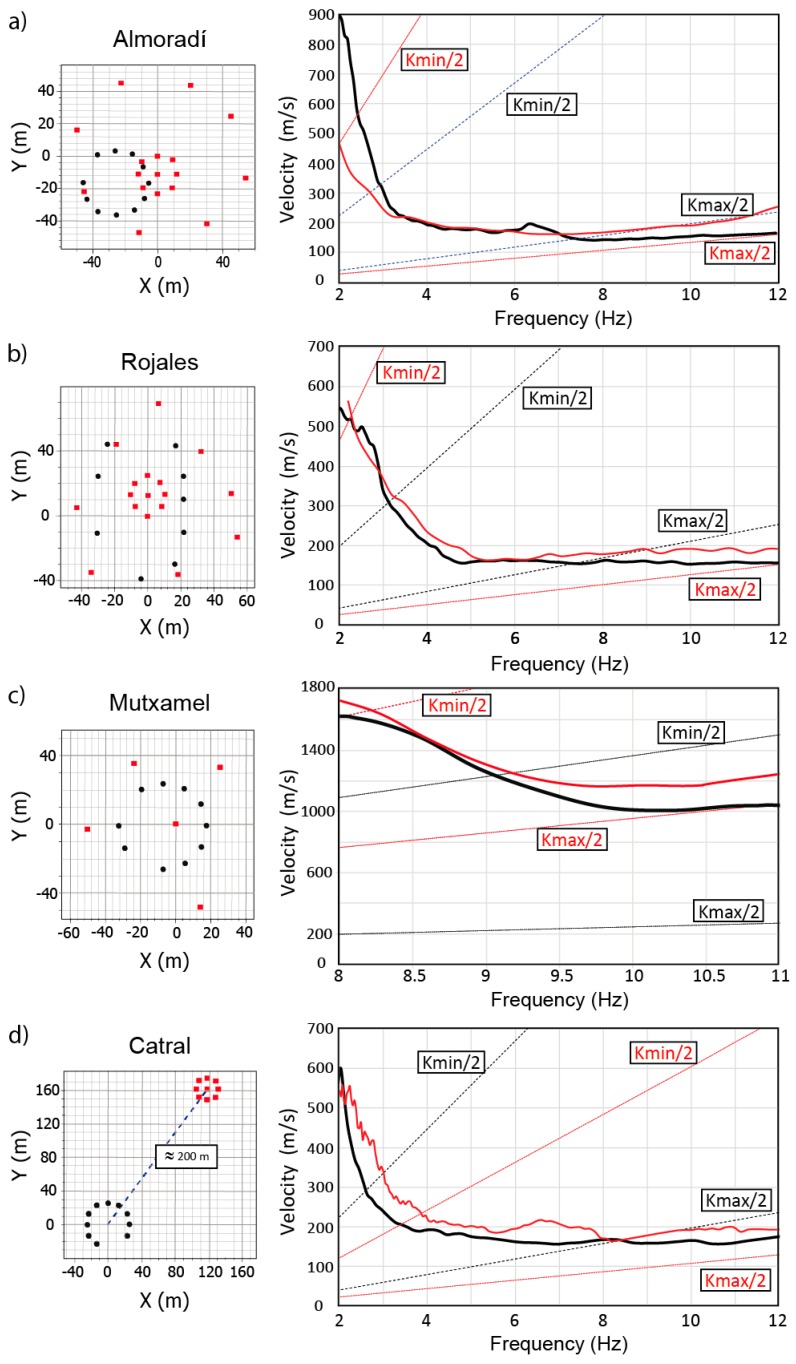
Array geometries (left column) and estimated dispersion curves (right column) for the Geophonino-W (black color) and the commercial equipment (red color) at the sites under study: (**a**) Almoradí; (**b**) Rojales; (**c**) Mutxamel; and (**d**) Catral. Wavenumber limit functions for kmax/2 and kmin/2 values are represented with dashed lines in the corresponding color.

The recorded signals were analyzed using the f-k method and the Geopsy software [52] and by applying the same configuration parameters used in previous studies. Thus, the recorded signals have been divided into 50 periods of frequency-dependent time length windows. In the wavenumber domain, the analysis is determined by the grid step and the grid size parameters, which are directly related to the minimum and maximum wavenumber limits, i.e., the kmax/2 and kmin/2 values, respectively. These limits are estimated from the theoretical array response and depend on the number of stations and their relative position in the array. Kmax provides information about the aliasing effects, while the kmin value establishes the resolution capability of the array to discriminate between waves travelling at close wavenumbers [53]. The theoretical wavenumber limits (kmax/2 and kmin/2), obtained for the different array configurations used in Almoradí, Rojales, Mutxamel and Catral (see Figure 19, left column), are shown in Table 2. In Figure 19, left column, the relative position of the stations for the measurements, carried out using Geophonino-W and the other commercial equipment, is indicated with square symbols and black dots, respectively. 

In order to test the reliability of the GPS locations, the relative position of the stations was obtained using a measuring tape and GPS data (Equation (1)). In this way, the coordinates estimated in both cases, as well as the corresponding dispersion curves obtained by the f-k analysis, were compared, without observing any significant difference between them. These results allow us to guarantee that the GPS locations provided by the developed system are accurate enough for the array analysis of seismic noise. It is important to note that, in a previous experimental work [54], the minimum precision required for the station positions within an array was established, with a maximum aperture of around 5%. Thus, in Almoradí, for example, where ten stations were deployed in a circular array, with a diameter of 40 m, the maximum allowed deviation in positions would be 2 m. In our case, the average deviations observed between the measuring tape and GPS estimated positions (Equation (1)) are lower than 1.5 meters.

In Figure 19, right column, the dispersion curves, estimated using the data recorded with Geophonino-W (black curves) and the other commercial equipment (red curves), are shown. Besides, the lines corresponding to the kmax/2 and kmin/2 limits are also displayed with the same color criterion as that of the dispersion curves.

In Almoradí (Figure 19a), two circular arrays of different apertures, 25 and 100 m, were deployed using the commercial equipment. Nine stations were used for each array, one of which was in the center of the circular layout, and eight were distributed around it. The complete dispersion curve was obtained, combining the results of both arrays. In the case of Geophonino-W, ten stations, deployed in a circular array, with a diameter of 40 m, were used. The obtained dispersion curves are very well matched in the range of 3.2–6.1 Hz. Small differences near the resolution and the aliasing limits of the curve are observed (3–3.2 Hz and 6.1–7 Hz, respectively). The mean relative difference between curves is 5.6%, measured in the valid frequency range of 3–7 Hz. These differences could be associated with the differences between the geometries and positions of the arrays.

The results obtained in the Rojales municipality are shown in Figure 19b. In this case, the complete dispersion curve, estimated using the commercial equipment, was obtained, combining the results from a circular array, with a diameter of 25 m, and a polygonal array, with a maximum distance between stations of around 100 m. For Geophonino-W, an ellipsoidal shaped geometry was deployed, with a major radius of 43 m and minor radius of 26 m. The selection of this geometry was conditioned by the available free area, which was different from what it was like in 2011. The obtained curves show a very good agreement in the valid range of 3–8 Hz. In this range, the measured mean relative difference is 7.3%. The shape of the curve is the same, with slight differences of 30–40 m/s in the range of 3–5 Hz.

The obtained dispersion curves for the Mutxamel site are shown in Figure 19c. This site has been selected, because the soil characteristics are very different from those of the other ones. It is practically located on rock. In this case, a circular array, with a diameter of 50 m and 10 stations, was deployed using Geophonino-W. For comparison with the commercial equipment, we used the results obtained in 2007, with an array of 5 sensors and maximum aperture of approximately 100 m. Both dispersion curves are well matched, but the one obtained with the commercial equipment seems to be affected by the aliasing effects for frequencies above 9.5 Hz. Despite this, the mean relative difference measured in the range of 8–11 Hz is 7.7%. In this case, the absence of short distances between stations leads to the appearance of aliasing effects. It should be noted that the minor distance between stations is 42 m in this geometry. This effect, in combination with the low number of stations used, lead us to think that the dispersion curve estimated using Geophonino-W offers a greater reliability than the one obtained by the commercial equipment.

Finally, the results obtained for the Catral site are shown in Figure 19d. The array deployed using the commercial equipment consisted of nine 10 Hz vertical geophones: one in the center, and the other eight distributed along a circumference, with a diameter of 25 m. In the case of the developed acquisition system, a circular array, with a diameter of approximately 40 m and ten 1 Hz sensors, was implemented. The place where the data were recorded in 2019 with Geophonino-W is separated by around 200 meters from the site, where the data were collected in 2014, because it was not possible to access the exact place where the data were taken in this year. The obtained curves do not show significant differences in the common valid range of 3.5–8.2 Hz. Both curves present the same behavior, although the velocities estimated using Geophonino-W are lower than the ones obtained using the commercial equipment, which is reflected in the mean relative difference of 13.9%, measured in the common valid range. These differences may be due to several factors: the use of different kinds of sensors (1 Hz and 10 Hz); the small aperture (only 25 m) of the array used to obtain the reference dispersion curve; and also the distance between the positions of the old array and the new one, which implies possible differences in the geological characteristics of the site. 

Among all of the methods included in the literature, we have chosen the f-k method, because this was the technique applied in previous works for the selected sites. Therefore, in order to validate the developed system, the results obtained using the commercial equipment have been compared with the ones obtained using Geophonino-W, demonstrating its suitability for seismic noise recording. In this way, the analysis of the data recorded by the developed system could be carried out not only using the f-k technique, but also using any other seismic noise analysis technique. 

## 5. Conclusions

In this paper, a wireless multichannel seismic noise recorder, Geophonino-W, was presented. The developed system allows array measurements to be carried out, without the use of cables. All of the hardware components, as well as the software design, were described in detail. The equipment was built exclusively using open-source hardware and software. Therefore, the configuration files, source code, PCB design and enclosure design were published in an open-source format in order to simplify the reproduction of the equipment. Furthermore, a developed GUI was provided, which guides the user through the data acquisition process. 

The remote modules of the Geophonino-W system are equipped with an anti-aliasing filter and an instrumentation amplifier, which allows for two amplifications (i.e., 34 dB (×50) and 20 dB (×10)), selectable by a switch. The modules also include two variable-gain amplifiers, whose combined gain varies from 0 dB (×1) to 52 dB (×400). Each of the remote stations is equipped with a GPS device, which is used to provide an estimation of its position. In this way, it is not necessary to manually measure the inter-station distances in the field.

Several laboratory experiments were conducted to characterize and evaluate the performance of the developed system. The tests carried out were related to the frequency response, precision, accuracy and battery duration of each of the remote stations, as well as the synchronization among stations. All of the obtained results prove the proper functioning of the Geophonino-W system.

In order to demonstrate the suitability of the system for seismic noise array measurements, four field campaigns were carried out at sites with different soil characteristics. Subsequently, the f-k analysis was applied to the recorded data, and the corresponding dispersion curves were obtained. In order to assess the performance of the Geophonino-W system, the obtained results were compared with the dispersion curves, estimated in other previous studies using commercial equipment. The comparison revealed a good agreement between all of the analyzed cases.

The calculation of the dispersion curve allows the corresponding shear-wave velocity profile associated with the site under study to be estimated, which is mandatory for determining the local effects of possible earthquakes. In general, the commercial equipment available in the market has a high price, which limits the application of these array techniques, especially in places with few economic resources. The developed Geophonino-W system is an economic option for small research groups or universities that want to carry out these array studies but lack the financial resources necessary to acquire wireless commercial equipment.

## Figures and Tables

**Table 1:** Technical specifications of the Arduino Due board.

Description	Value/Range
Operating Voltage	5 V
Input Voltage (recommended)	7–12 V
Input Voltage (limits)	6–16 V
Digital I/O Pins	54 (of which 12 provide PWM output)
Analog Input Pins	12
Analog Output Pins	2 (DAC)
Total DC Output Current on al I/O lines	130 mA
DC Current for 3.3 V and 5 V Pin	800 mA
Flash Memory	512 Kilobytes (KB)
SRAM	96 KB (two banks: 64 KB and 32 KB)
Clock Speed	84 MHz
Length	101.52 mm
Width	53.3 mm
Weight	36 g.

**Table 2:** Theoretical wavenumber limits (rad/m) for the different geometries used.

	Commercial equipment		Geophonino-W	
Site	Kmax/2	Kmin/2	Kmax/2	Kmin/2
Almoradí	0.470	0.027	0.319	0.056
Rojales	0.500	0.027	0.299	0.063
Mutxamel	0.065	0.031	0.255	0.046
Catral	0.533	0.096	0.319	0.056

## Supplementary Materials

The following are attached to this article: The source code of ACN, MCN and GUI; configuration files for Xbee modules; PCB design in a Gerber file format; enclosure designs in an stl format; recoded data for Section 3: System characterization and tests, which is available online at https://www.mdpi.com/1424-8220/19/19/4087/s1.
